# An investigation for the efficacy of teaching model of combining virtual simulation and real experiment for clinical microbiology examination

**DOI:** 10.3389/fmed.2024.1255088

**Published:** 2024-02-21

**Authors:** Ling Meng, Xia Liu, Jing Ni, Pei Shen, Fengping Jiao

**Affiliations:** School of Public Health, Shandong First Medical University and Shandong Academy of Medical Sciences, Jinan, Shandong Province, China

**Keywords:** virtual simulation, clinical microbiology examination, experimental teaching model, clinical thinking, experimental skill

## Abstract

**Background:**

As a convenient teaching tool, virtual simulation experiment technology had been widely utilized in the field of medical education. However, virtual learning could not fully replace the benefits of in-person instruction. Therefore, finding ways to integrate both methods was crucial for achieving optimal educational outcomes. The objective of this study was to compare the effectiveness of the self-built virtual simulation and design experiment combining teaching mode and the traditional experimental teaching mode in the clinical microbiology examination experiment teaching.

**Methods:**

This study was conducted at Shandong First Medical University in China. The experimental group consisted of 100 third-year students from the grade 2020 majoring in medical examination technology, who underwent an innovative teaching model combining virtual and real experiments. The control group comprised of 100 third-year students from the grade 2019 in the same major, who received traditional experimental teaching model. In this study, we referred to grade 2020 as cohort 2020 and grade 2019 cohort 2019. The performance of both groups was assessed via experimental and theoretical testing. Meanwhile, survey questionnaires were administered to evaluate the efficacy of the innovative experimental teaching model and students’ level of satisfaction with it. Cohort 2020 conducted a survey for modules 1 to 4, while cohort 2019 only conducted a survey for module 4, as detailed in the Appendix.

**Results:**

The majority of students in the experimental group expressed satisfaction with the teaching model that combined virtual and real experiments, as evidenced by their superior performance on both experimental operational skills (87.54 ± 8.93 vs. 82.39 ± 10.55) and theoretical knowledge tests (83.65 ± 9.02 vs. 80.18 ± 8.24) compared to those in the control group.

**Conclusion:**

The combination of virtual simulation experiment and design experiment in the microbiological examination of clinical specimens represented an effective pedagogical approach. The instructional approach had the potential to incite a passion for learning, enhance proficiency in standardized experimental techniques, foster the ability to integrate theory with practice, and cultivate clinical reasoning skills.

## 1 Introduction

Clinical microbiology examination was a specialized course for students majoring in medical examination technology that primarily focused on the biological characteristics of pathogenic *microorganisms* and methods for microbial examination (1, 2). The theoretical knowledge of this course provided the necessary evidence for the diagnosis of diseases associated with microbial infections, which was important for detecting pathogenic microorganisms using standardized experimental techniques (3). However, challenges existed in the course. Firstly, students had insufficient time to practice and consolidate their learning from the class due to limited hours allocated for practical sessions. Secondly, the traditional experimental teaching approach was primarily focused on verification experiments, which posed a challenge for students to effectively integrate theory with practice and enhance their practical skills. Thirdly, the biosafety regulations of the laboratory made it impossible to detect pathogenic *microorganisms*, which restricted students’ ability to detect such *microorganisms*.

It was acknowledged that virtual simulation technology was a convenient and effective tool. The virtual simulation experiment was designed to meet the objectives of experimental teaching and replicate real-world experimental environments (4, 5). Students engaged in immersive and realistic virtual experiments through human-computer interaction, and acquired necessary skills for independent practice when learners responded in what they perceived as realistic (6, 7).

However, the virtual simulation technology had the limitation that it could not be operated in real experiment (8). Therefore, it was of paramount importance to integrate virtual simulation experiments with actual ones and fully leverage the benefits of the former (9–11). The substitution of virtual for real could compensate for experiments that could not be conducted due to biosafety concerns or other reasons, while combining both approaches could effectively broaden and deepen experimental teaching.

This study aimed to compare the effectiveness of the self-built virtual simulation and design experiment combining teaching mode and the traditional experimental teaching mode in the clinical microbiology examination experiment teaching.

## 2 Research methods

### 2.1 Object of study

The study received approval from the Research and Ethics Committee of Shandong First Medical University. 100 third-year students enrolled in 2019 majoring in medical examination technology served as the control group and received conventional teaching methods without virtual simulation experimental platform training, offline discussion, and design experiment for microbiological examination of clinical specimens. The experimental group consisted of 100 third-year students enrolled in 2020 majoring in medical examination technology received these additional training methods named “the integration of virtual and reality “. To provide equal opportunity for both control and experimental groups with both learning opportunities, the virtual simulation experiment website was published to guide the students of cohort 2019 to carry out virtual simulation experiment after collecting the data for this study. In addition, offline discussion and design experiment were given in spare time.

### 2.2 Teaching strategies

**Figure 1:**
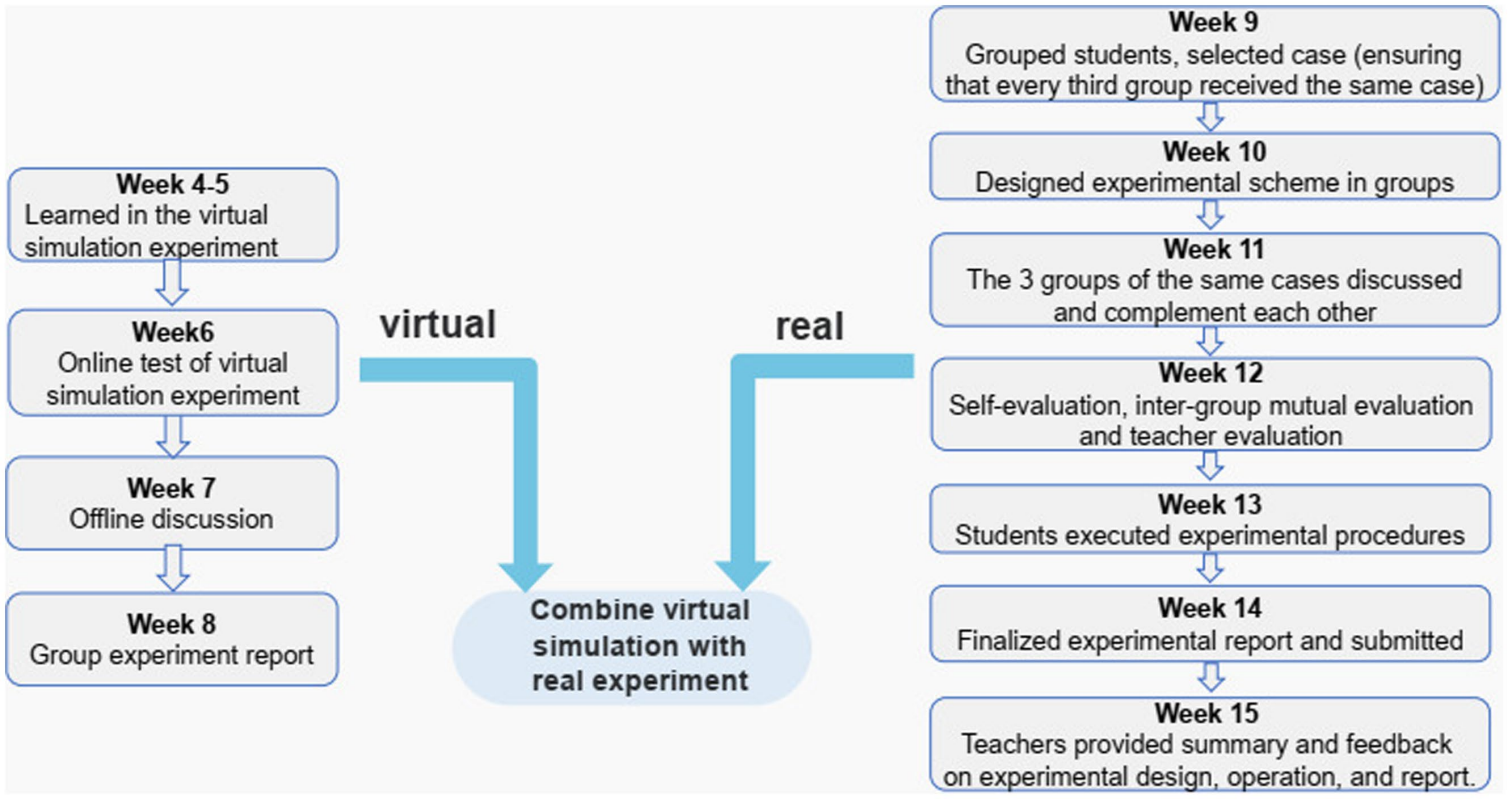
Flow chart of the teaching model of combining virtual simulation and real experiment.

The experimental instructional design consisted of two parts of virtual simulation experiment operation and real experiment, as illustrated in Figure 1. Both groups received theoretical and experimental training from the same teacher, respectively.

#### 2.2.1 Virtual simulation experiment operation

##### 2.2.1.1 Construction of the virtual simulation experiment

An innovative virtual simulation experiment named “Detection of *Vibrio cholerae*” was developed based on current challenges in experimental teaching of clinical microbiology examination. This virtual simulation covered the clinical significance, laboratory detection and results of *Vibrio cholerae*. The laboratory detection was comprised of 13 microbiology test experiments, including morphological examination, isolation culture, biochemical reaction, drug sensitivity testing and serological testing of bacteria. It covered nearly all the experimental operation method of the course. More importantly, the experimental operation method of the self-created virtual simulation experiment was standardized and scientific, which effectively enhanced students’ proficiency in conducting standardized microbiological experiments.

##### 2.2.1.2 Design concept of self-created virtual simulation experiment

The diagnosis and treatment process of cholera patients was taken as the main focus throughout the entire virtual simulation experiment. It simulated the microbiology diagnostic working process from specimen reception and processing to laboratory examination, result report, and emergency treatment of cholera patients in a clinical laboratory, which closely integrated experimental teaching with clinical practice to cultivate students’ clinical thinking. The interest in learning of student could be enhanced by presenting a doctor-patient dialogue that covered the symptoms, prevention and prognosis, as well as emergency treatment of cholera and the integration of the theory and practice. Additionally, an animated presentation on the classification, transmission route, pathogenic mechanism and clinical manifestations of *Vibrio cholerae* provided a multi-dimensional stimulation for students to achieve better learning outcomes.

##### 2.2.1.3 Learning process of virtual simulation experiment

Students logged in the virtual simulation experiment using their individual login credentials, where they were introduced to the clinical significance of Cholera before proceeding to engage with two distinct modules: “guidance” and “assessment.” The former provided a detailed breakdown of each experimental step, allowing beginners to follow along and learn through practical application. Students could move on to the latter module and conduct their own assessments after getting comfortable with the process. The system evaluated students’ operational proficiency to assess their learning outcomes. The “guidance” and “assessment” modules could be interchanged and repeated until all knowledge points were fully grasped. The result-oriented teaching approach were promoted to encourage student to learn actively.

##### 2.2.1.4 The learning arrangement of virtual simulation experiment

Students were instructed to commence the virtual simulation experiment during the fourth week of instruction, followed by an online assessment in teaching week 6. In teaching week 7, teachers and students jointly participated in offline case discussions regarding *Vibrio cholerae* identification. In teaching week 8, students completed group-experimental reports on *Vibrio cholerae* identification, which systematically honed their clinical thinking skills. In teaching week 9, the teacher corrected the experiment reports and gave feedback to students.

#### 2.2.2 Design experiment for microbiological examination of clinical specimens

##### 2.2.2.1 Collect clinical cases and prepare specimens

The three clinical cases were presented in Table 1, and simulated clinical specimens had been prepared beforehand.

**Table 1:** The case information for clinical specimen design experiment.

Case number	Case information
Case 1	The patient, a 55-year-old male, was admitted with high fever and chills. He presented sudden onset of chest pain and purulent sputum. The result of X-rays revealed that he suffers from the necrotizing pneumonia. His general condition was poor, likely due to the diagnosis of diabetes before 10 years. Sputum samples were collected for microbiological examination.
Case 2	The patient, a 61-year-old female, was admitted to the hospital presenting with cough, phlegm and wheezing. Symptoms had a slow onset with marked morning cough producing frothy or serous sputum that appeared bluish-green in color. The general condition of the patient was poor with coarse breath sounds and feverishness. Sputum samples were collected for microbiological examination.
Case 3	The patient, a 14-year-old male, was admitted with symptoms of abdominal pain and fever characterized by the sudden onset, persistent severity, rotation, deep breathing exacerbation upon coughing. Tenderness and rebound pain were observed in the right lower abdomen. Abdominal puncture yielded mixed blood fluid for etiological examination.

##### 2.2.2.2 Organize students into groups

The 100 students were divided into three laboratories, with each lab consisting of nine groups with three to four students. The cases were randomly selected and evenly distributed among the groups, ensuring that every third group received the same case. Each laboratory was staffed by a trained teacher for guided laboratory operations, all of whom followed the same teaching program.

##### 2.2.2.3 Conduct experimental design

According to the selected cases, students were grouped to complete the experimental program design for pathogenic *microorganisms’* detection of clinical specimens during the 10th teaching week. One week later, the 3 student groups were assigned to the same case discussed and complement each other, reached a consensus, and improved and perfected experimental program design, then all perfected experimental program designs were submitted to the Chinese University MOOC platform. In the 12th teaching week, the group self-evaluation, inter-group mutual evaluation (according to the scoring scale) and teacher evaluation were completed online within 3 days, and on days 4–5, teachers provided feedback on the experimental program designs, and on days 6–7, students made improvements based on this feedback and resubmitted their work online.

##### 2.2.2.4 Perform experimental operation

In the 13th week of instruction, students executed experimental procedures based on the aforementioned improved design programs and completed pathogenic *microorganisms’* detection in simulated clinical specimens. The instructor provided appropriate guidance.

##### 2.2.2.5 Write the experimental report

In the 14th teaching week, the experimental report must be finalized and submitted online. During the 15th week, group self-evaluation, inter-group mutual evaluation and teacher evaluation based on the rating scale were completed.

##### 2.2.2.6 Summary and feedback

In the 15th teaching week, teachers provided a summary and feedback on experimental program design, operation, and experimental report.

## 3 Student assessment

Student assessment comprised laboratory test, theoretical test, and questionnaires administered after the teaching. The laboratory test aimed to evaluate students’ proficiency in experimental techniques such as gram staining, oxidase testing, and catalase testing. The theoretical test was conducted in a closed-book format that assessed basic theoretical knowledge and clinical case analysis ability. The small program named Questionnaire Star was used for questionnaire survey.

### 3.1 Statistical analysis

The experimental and theoretical test scores of both the control and experimental groups were inputted into SPSS 25.0 software (SPSS Inc., Chicago, Illinois, United States). The data was presented as means ± standard deviations. Independent t-test was used for continuous variables such as age and test scores. Chi-square test was employed for categorical variables such as sex (male/female). A significance level of *p* < 0.05 was utilized.

## 4 Results

### 4.1 Comparison of course grades between the two groups

The 100 third-year students from the cohort 2020 majoring in medical examination technology were assigned to the experimental group, and 100 third-year students from the cohort 2019 majoring in medical examination technology were assigned to the control group. The two groups were comparable in terms of age and sex distribution (Table 2). Results of course grades showed that students in the experimental group outperformed those in the control group on both practical skills test (87.54 ± 8.93 vs. 82.39 ± 10.55) and theoretical test (83.65 ± 9.02 vs. 80.18 ± 8.24) at the end of the term, as presented in Table 3.

**Table 2:** The basic information of students in experimental cohort 2020 and control cohort 2019.

		Experimental group (*n* = 100)	Control group (*n* = 100)	*t/* $\chi^{2}$ -value	*p* -value
	Age	21.07 ± 1.35	21.31 ± 1.29	1.285	0.200
	Sex				
	Female [*n* (%)]	64 (64.0)	61 (61.0)		
	Male [*n* (%)]	36 (36.0)	39 (39.0)	0.192	0.661

**Table 3:** Comparison of the course grades of theoretical test and experimental skills test between students in experimental cohort 2020 and control cohort 2019.

	Experimental group (*n* = 100)	Control group (*n* = 100)	*t* value	*p* value
theoretical test	83.65 ± 9.02	80.18 ± 8.24	2.840	0.005
experiment skills test	87.54 ± 8.93	82.39 ± 10.55	3.726	<0.001

### 4.2 Questionnaire results of virtual simulation experiment in experimental group

At the end of the term, one hundred questionnaires regarding virtual simulation experiment were distributed to students in the experimental group. All questionnaires were retrieved, resulting in a 100% recovery rate (Table 4). The result of the questionnaire survey indicated that our self-created virtual simulation experiment for *Vibrio cholerae* detection had provided a highly effective learning experience. The survey results showed that 90% of students thought that the virtual simulation experiment was easy to understand, and approximately 78% of students acknowledged that the navigation and instructions offered by the program facilitated their understanding. Additionally, the survey results displayed that 90% of students praised the video and audio quality of this virtual simulation experiment. The results also turned out that 92% of the students perceived virtual simulation experimental examination as more equitable and objective than traditional experimental examinations, and almost 89% of students believed that virtual simulations were beneficial for both practical operation learning and theoretical knowledge consolidation. Furthermore, results of the survey revealed that 75% of the students felt that virtual simulations provided a lifelike experience with better time management, and they also reported feeling actively engaged in the process. In the end, about 85% of the students expressed satisfaction with the virtual simulation experiment.

**Table 4:** Results of a questionnaire on the learning experience in virtual simulation experiments of the students in experimental cohort 2020.

Question	Agree *n* (%)	Fall in between *n* (%)	Disagree *n* (%)
1. The virtual simulation experiment proved to be user-friendly.	90	2	8
2. The navigation of the virtual simulation experiment was simple and clear.	78	13	9
3. It is easy to learn the virtual simulation experiment according to the provided instructions.	78	15	7
4. The virtual simulation experiment boasted exceptional video and audio quality.	90	10	0
5. The evaluation of virtual simulation is more equitable and objective compared to traditional experimental assessment.	92	7	1
6. The virtual simulation experiment was a valuable tool for enhancing practical skills and reinforcing theoretical knowledge in experimental operations.	89	9	2
7. The virtual simulation experiment provided a lifelike experience.	75	20	5
8. The management of time could be optimized through virtual simulation experiments.	75	18	7
9. I was more actively engaged in the virtual simulation experiment compared to the traditional experimental classes.	70	25	5
10. I was completely satisfied with virtual simulation experiment.	85	15	0

### 4.3 Questionnaire results of a design experiment for microbiological examination of clinical specimens in experimental group

In the same way, the design experiment questionnaire for microbiological examination of clinical specimens in the experimental group was completed with a 100% recovery rate, similarly as shown in Table 5. The survey results indicated that 85% of the students were able to complete their team tasks within the given timeframe, and approximately 78% of the students believed that timely feedback from both group members and teachers was beneficial in enhancing their learning experience. Furthermore, there were 81% of the students reported an increase in interest towards learning as a result of the participation in the experiment. Moreover, survey results displayed that 89% of the students noted that all team members actively participated during the experiment. Finally, up to 90% of the students expressed pride in independently completing pathogenic *microorganisms’* detection on clinical specimens.

**Table 5:** Results of a questionnaire for a design experiment of the microbiological examination of clinical specimens of experimental cohort 2020.

Question	Agree *n* (%)	Fall in between *n* (%)	Disagree *n* (%)
1. Could you complete your team tasks on time?	85 (85.0)	10 (10.0)	5 (5.0)
2. Had your group members and teachers provided timely and effective feedback to enhance your learning experience?	78 (78.0)	12 (12.0)	10 (10.0)
3. Would this experiment enhance your learning motivation?	81 (81.0)	16 (16.0)	3 (3.0)
4. Did the participants in your group actively participate in the experiment?	89 (89.0)	9 (9.0)	2 (2.0)
5.Were you proud of independently completing the detection of pathogenic *microorganisms* in clinical specimens?	90 (90.0)	8 (8.0)	2 (2.0)
6.Would this experiment acquaint you with the workflow of the clinical microbiology laboratory?	90 (90.0)	7 (7.0)	3 (3.0)

### 4.4 Questionnaire results of virtual and real experiment combined teaching method in experimental group

Table 6 presented the results of a questionnaire assessing the effectiveness of the combination virtual and real experiments in teaching Clinical Microbiology Examination. The majority (90%) of students expressed satisfaction with the learning resources provided by virtual simulation experiments and clinical specimen examinations, while 84% agreed that the combination of such simulation and offline discussion facilitated the completion of microbiological examinations. Additionally, there were 78% of the students were satisfied with the experimental design scheme that integrated virtual simulation and microbiological examination of clinical specimens. More importantly, the results showed that 89% of the students believed that this combination facilitated their understanding of microbiological testing concepts for clinical specimens, enhanced their clinical thinking abilities, and proved to be an effective teaching method in Clinical Microbiology Examination.

**Table 6:** Questionnaire results of virtual and real experiment combined teaching method of experimental cohort 2020.

Question	Agree *n* (%)	Fall in between *n* (%)	Disagree *n* (%)
1.Were the learning resources provided by the virtual simulation experiment and microbiological examination of clinical specimens satisfactory to you?	90 (90.0)	5 (5.0)	5 (5.0)
2. Did the utilization of virtual simulation experiment and offline discussion contribute to the successful completion of microbiological examination on clinical specimens?	84 (84.0)	12 (12.0)	4 (4.0)
3. Were you satisfied with the experimental teaching approach that integrated virtual simulation and microbiological examination of clinical specimens?	78 (78.0)	16 (16.0)	6 (6.0)
4. Did virtual simulation and microbiological examination of clinical specimens help you understand the ideas for microbiological testing of clinical specimens?	89 (89.0)	9 (9.0)	2 (2.0)
5. Did you agree that the teaching method of virtual and real experiment combined can improve clinical thinking and the ability to combine theory with practice?	89 (89.0)	7 (7.0)	4 (4.0)
6. Did you think that the combine of virtual and real experiment is an effective teaching method in clinical microbiology examination?	89 (89.0)	10 (10.0)	1 (1.0)

### 4.5 Questionnaire results of learning effectiveness satisfaction levels of two student groups

Table 7 presented the results of a questionnaire on learning effectiveness satisfaction levels of two student groups. A total of 200 questionnaires were distributed and returned, resulting in a 100% response rate. The survey findings indicated that the experimental group reported higher levels of satisfaction than the control group with respect to “keen interest in learning,” “rudimentary knowledge acquisition,” “standardized experimental techniques,” “development of clinical thinking skills,” “integration of theory and practice,” “self-directed learning improvement,” and “strengthening communication skills,” the difference was statistically significant (*p* < 0.05).

**Table 7:** The comparisons of the learning effectiveness satisfaction levels of experimental cohort 2020 and control cohort 2019.

Question	Experimental group *n* (%)	Control group n (%)	$\chi^{2}$ -value	*p*-value
1. You demonstrated a keen interest in acquiring knowledge.	75 (75.0)	54 (54.0)	9.630	0.002
2. You grasped rudimentary knowledge of this course.	78 (78.0)	50 (50.0)	17.014	<0.001
3. Your technique for conducting experiments was executed in a standard manner.	85 (85.0)	61 (61.0)	14.612	<0.001
4. You had developed a certain level of clinical thinking.	77 (77.0)	46 (46.0)	20.294	<0.001
5. You had the ability to integrate theory with experiment.	77 (77.0)	46 (46.0)	20.294	<0.001
6. You improved your self-directed learning abilities.	81 (81.0)	63 (63.0)	8.036	0.005
7. You had enhanced your proficiency in communication.	80 (75.0)	45 (45.0)	26.133	<0.001

## 5 Discussion

In this study, the efficacy of teaching model of combining virtual simulation and real experiment for Clinical Microbiology Examination were studied. During the experiment, students in the experimental group performed a “the integration of virtual and reality “and online discussions, while the control group received only traditional teaching methods without above training. The questionnaires covered four aspects: the learning experience in virtual simulation experiment and a design experiment of the microbiological examination of clinical specimens, the effectiveness of virtual and real experiment combined teaching method, learning effectiveness satisfaction levels. And results were collected and analyzed. Results showed that experimental group students had higher scores in both practical skills test and theoretical test than the control. It indicated that the innovative “the integration of virtual and reality “improved students’ mastery of knowledge and skills. The survey results also reported that approximately 85 and 90% of the students expressed satisfaction with the virtual simulation experiment and the design experiment for microbiological examination of clinical specimens, respectively. More importantly, about 89% of the students believed that “the integration of virtual and reality “were helpful to the understanding and learning of the clinical microbiology examination. Above results could be attributed to the repetitive training provided by virtual simulation and students’ interests in design experiment (12–15). As virtual simulation could be considered for just-in-time training before exposure to traditional lab activities, for specific skill acquisition using deliberate practice (16, 17). Therefore, by integrating virtual and realistic experiments, the professional skills mastered by learners in the virtual simulation environment could be applied to specific practice, which could effectively improve the understanding of knowledge. Results from the learning effectiveness satisfaction survey on two groups of students showed that compared to the control group, the experimental group exhibited stronger learning motivation, higher professional competence, and enhanced clinical thinking ability as well as improved capacity in integrating theory with practice. These findings aligned with prior research highlighting higher student satisfaction with virtual simulations (18, 19).

Compared with previous studies (19–22), our approach incorporated offline discussion on the basis of virtual simulation, and more importantly, combined virtual simulation with real experiment. The microbiological detection experiment of clinical specimens was carried out after further sorting out the knowledge, skills learned from the virtual simulation experiment and clinical thinking of microbial detection through offline discussions, which enabled the conversion of theory into practice and solved the problem of converting virtual simulation into actual operation to a certain extent. However, there were limitations in this study. First, because there was only one teaching class of medical examination technology majored in our school each year, the control group in this study could only use the learning data of cohort 2019 students to conduct quasi-experimental research, rather than experimental research, which might cause some bias in the results. Second, the teaching reform had only been tried in one teaching class and needed to be carried out several more times to collect more data to ensure the reliability of the results.

In summary, this study adopted a results-oriented approach to address teaching challenges and enhance students’ learning abilities. By integrating virtual and real experiments and leveraging their respective advantages, it motivated learners, improved proficiency in standardized experimental techniques, fostered the integration of theory with practice, and cultivated clinical reasoning skills. Therefore, the pedagogical framework of combining virtual and real methods was highly effective, and it was worth popularizing and applying in similar courses. In the future, studies would be conducted on the attention of a single variable, such as designing experiments to explore which of the influencing factors plays a major role.

## Data availability statement

The original contributions presented in the study are included in the article/Supplementary material, further inquiries can be directed to the corresponding author.

## Ethics statement

The studies involving human participants were reviewed and approved by the Research and Ethics Committee of Shandong First Medical University (R202203010089). Written informed consent from the participants was not required to participate in this study in accordance with the national legislation and the institutional requirements.

## Author contributions

LM: Investigation, Methodology, Supervision, Validation, Visualization, Writing – review & editing. XL: Data curation, Investigation, Supervision, Validation, Visualization, Writing – review & editing. JN: Data curation, Software, Supervision, Validation, Writing – review & editing. PS: Data curation, Software, Supervision, Validation, Writing – review & editing. FJ: Conceptualization, Formal Analysis, Funding acquisition, Investigation, Project administration, Resources, Supervision, Validation, Writing – original draft, Writing – review & editing.
