# Supplementary material for: An investigation for the efficacy of teaching model of combining virtual simulation and real experiment for clinical microbiology examination
Source: Front Med (Lausanne). 2024 Feb 21;11:1255088. doi: 10.3389/fmed.2024.1255088 (PMC10915005; doi:10.3389/fmed.2024.1255088)
Supplement: Supplementary file 1 [file Data_Sheet_1.doc]

**Appendix**

**Questionnaire for clinical microbiology laboratory course learning**

**Kind reminder:**

1. This study was approved by the Research and Ethics Committee of Shandong First Medical University, and all personal information of students will be treated with confidentiality.
2. This questionnaire is for third-year students from the **cohort 2020** majoring in medical examination technology.

Module 1: questionnaire on the learning experience in virtual simulation experiments

| 1. The virtual simulation experiment proved to be user-friendly.   A. agree B. fall in between C. disagree |
| --- |
| 1. The navigation of the virtual simulation experiment was simple and clear.   A. agree B. fall in between C. disagree |
| 1. It is easy to learn the virtual simulation experiment according to the provided instructions.   A. agree B. fall in between C. disagree |
| 1. The virtual simulation experiment boasted exceptional video and audio quality. |
| A. agree B. fall in between C. disagree |
| 1. The evaluation of virtual simulation is more equitable and objective compared to traditional experimental assessment.   A. agree B. fall in between C. disagree |
| 1. The virtual simulation experiment was a valuable tool for enhancing practical skills and reinforcing theoretical knowledge in experimental operations.   A. agree B. fall in between C. disagree |
| 1. The virtual simulation experiment provided a lifelike experience.   A. agree B. fall in between C. disagree |
| 1. The management of time could be optimized through virtual simulation experiments.   A. agree B. fall in between C. disagree |
| 1. I was more actively engaged in the virtual simulation experiment compared to the traditional experimental classes.   A. agree B. fall in between C. disagree |
| 1. I was completely satisfied with virtual simulation experiment.   A. agree B. fall in between C. disagree |

Module 2: questionnaire for a design experiment of the microbiological examination of clinical specimens

| 1. Could you complete your team tasks on time?   A. agree B. fall in between C. disagree |
| --- |
| 1. Had your group members and teachers provided timely and effective feedback to enhance your learning experience?   A. agree B. fall in between C. disagree |
| 1. Would this experiment enhance your learning motivation?   A. agree B. fall in between C. disagree |
| 1. Did the participants in your group actively participate in the experiment?   A. agree B. fall in between C. disagree |
| 1. Were you proud of independently completing the detection of pathogenic *microorganisms* in clinical specimens?   A. agree B. fall in between C. disagree |
| 1. Would this experiment acquaint you with the workflow of the clinical microbiology laboratory?   A. agree B. fall in between C. disagree  **Module 3: questionnaire of virtual and real experiment combined teaching method** |
| 1. Were the learning resources provided by the virtual simulation experiment and microbiological examination of clinical specimens satisfactory to you?   A. agree B. fall in between C. disagree |
| 1. Did the utilization of virtual simulation experiment and offline discussion contribute to the successful completion of microbiological examination on clinical specimens?   A. agree B. fall in between C. disagree |
| 1. Were you satisfied with the experimental teaching approach that integrated virtual simulation and microbiological examination of clinical specimens?   A. agree B. fall in between C. disagree |
| 1. Did virtual simulation and microbiological examination of clinical specimens help you understand the ideas for microbiological testing of clinical specimens?   A. agree B. fall in between C. disagree |
| 1. Did you agree that the teaching method of virtual and real experiment combined can improve clinical thinking and the ability to combine theory with practice?   A. agree B. fall in between C. disagree |
| 1. Did you think that the combine of virtual and real experiment is an effective teaching method in clinical microbiology examination?   A. agree B. fall in between C. disagree |

**Module 4: questionnaire of** the learning effectiveness satisfaction

| 1. You demonstrated a keen interest in acquiring knowledge.   A. agree B. fall in between C. disagree |
| --- |
| 1. You grasped rudimentary knowledge of this course.   A. agree B. fall in between C. disagree |
| 1. Your technique for conducting experiments was executed in a standard manner.   A. agree B. fall in between C. disagree |
| 1. You had developed a certain level of clinical thinking.   A. agree B. fall in between C. disagree |
| 1. You had the ability to integrate theory with experiment.   A. agree B. fall in between C. disagree |
| 1. You improved your self-directed learning abilities.   A. agree B. fall in between C. disagree |
| 1. You had enhanced your proficiency in communication.   A. agree B. fall in between C. disagree |

**Questionnaire for clinical microbiology laboratory course learning**

**Kind reminder:**

1.This study was approved by the Research and Ethics Committee of Shandong First Medical University, and all personal information of students will be treated with confidentiality.

2.This questionnaire is for third-year students from the **cohort 2019** majoring in medical examination technology.

**Module 1: questionnaire of** the learning effectiveness satisfaction

| 1. You demonstrated a keen interest in acquiring knowledge.  A. agree B. fall in between C. disagree |
| --- |
| 2. You grasped rudimentary knowledge of this course.  A. agree B. fall in between C. disagree |
| 3. Your technique for conducting experiments was executed in a standard manner.  A. agree B. fall in between C. disagree |
| 4. You had developed a certain level of clinical thinking.  A. agree B. fall in between C. disagree |
| 5. You had the ability to integrate theory with experiment.  A. agree B. fall in between C. disagree |
| 6. You improved your self-directed learning abilities.  A. agree B. fall in between C. disagree |
| 7. You had enhanced your proficiency in communication.  A. agree B. fall in between C. disagree |
