# Supplementary material for: An investigation for the efficacy of teaching model of combining virtual simulation and real experiment for clinical microbiology examination
Source: Front Med (Lausanne). 2024 Feb 21;11:1255088. doi: 10.3389/fmed.2024.1255088 (PMC10915005; doi:10.3389/fmed.2024.1255088)
Supplement: Supplementary file 2 [file Data_Sheet_2.pdf]

Ethics Review Committee of Shandong First Medical University

Ethical approval documents for biomedical research projects involving humans

No.（R202203010089）

<table>
<tr><td>Project name</td><td colspan="5">Research on clinical microbiology laboratory teaching practice based on OBE concept</td></tr>
<tr><td>Project name</td><td colspan="5">foundation medicine √   clinical medicine   medicine</td></tr>
<tr><td>Project funding</td><td colspan="3">Education Department of Shandong Province</td><td>Expenditure</td><td>$50000</td></tr>
<tr><td>Applicant</td><td colspan="3">Shandong First Medical University</td><td>Key Personnel</td><td>Xiaomei Li</td></tr>
<tr><td>Research Department</td><td>School of Public Health and Health Administration</td><td>Project Leader</td><td>Fengping Jiao</td><td>Job title</td><td>associate professor</td></tr>
</table>

Ethical review opinion	
∆ Agree	√
∆ Disagree (project termination or suspension)	
∆ Revise and review	

Approval opinion

The Ethics Committee conducted an ethical review on the project of "Research on classroom teaching Practice of Clinical Microbiology Laboratory based on the concept of OBE" After accepting application of Fengping Jiao. The committee concluded that the project conformed to the principle of medical purpose ethics and did not harm the subjects. In the study, the informed consent of the subjects was obtained in written form, which was in line with the principles of no harm and informed consent. The design of the research project basically conformed to international medical ethics documents such as the Declaration of Helsinki and relevant laws, regulations and ethical requirements of China.

The researchers also undertook to abide by the principles set forth by the World Medical Association WMA through the Declaration of Helsinki, respect the ethical recommendations made by the Ethics Committee for the study of this project and protect the personal privacy of the subjects. All the original data and related documents were kept in confidential archives for at least three years after the end of the study. All data were saved during the study for review and summary.

This committee certified that the Research on classroom teaching Practice of Clinical Microbiology Laboratory based on the concept of OBE" basically meets the ethical requirements.

Ethics Review Committee of Shandong First Medical University

（Seal）

March 1, 2022
